# Retraction Note: A Mathematical Model for Vibration Behavior Analysis of DNA and Using a Resonant Frequency of DNA for Genome Engineering

**DOI:** 10.1038/s41598-022-15829-9

**Published:** 2022-07-04

**Authors:** Mobin Marvi, Majid Ghadiri

**Affiliations:** Faculty of Engineering, Department of Mechanics, Imam Khomeini International University, P.O. Box 34149-16818, Qazvin, Iran

Retraction of: *Scientific Reports* 10.1038/s41598-020-60105-3, published online 26 February 2020

The Editors have retracted this Article. Following publication, major concerns were raised in regard to the lack of validation (against other models as well as experimentally). Several statements are unsupported, including the main conclusion that DNA in cancerous cells loses its ability for proteinization during DNA resonance, and therefore DNA resonance may be applied to control cancer. Moreover, the feasibility of its application in cancer treatment is unclear given the lack of specificity. The Editors therefore no longer have confidence in the conclusions of this Article.

Mobin Marvi and Majid Ghadiri do not agree to this retraction.

